# Phosphor-Necrosis

**Published:** 1865-10

**Authors:** 


PHOSPHOR-NECROSIS. — The deleterious effects of white phosphorus upon the health of the workmen engaged in making lucifer matches, have long attracted the attention of medical men. In 1845, Dr. Lorinser, of Vienna, published nine cases of necrosis in the jaw bone, five of which terminated fatally. At Lyons, the Commission of Public Health, some years ago, discovered twelve similar cases, and ascertained that one workman out of ten was liable to be attacked with that affection. In 1847, M. Shretter discovered that phosphorus, at a certain temperature, acquired different properties, such as a change of color from white to red, the loss of its peculiar smell of garlic, and that of shining in the dark. These characteristics of red or amorphous phosphorus, so different from those of the white kind, induced M. Chevallier to try whether it might not be advantageously used in the manufacture of matches. Some experiments made by Dr. Bussy at the same time proving the innocuousness of red phosphorus, taken internally, it only remained to be seen whether, combined with chlorate of potash, it would catch fire; and this having been satisfactorily ascertained, there is now no reason why the use of white phosphorus should not be prohibited by law, as is now proposed by Dr. Tunel. He states that workmen engaged in the manufacture of lucifer matches, with white phosphorus, soon experience difficulty of digestion, sore throat, and a bad cough. If they continue in the same trade after these symptoms they are exposed to the mortification of the jaw-bones, chiefly the lower one. This disease generally commences with toothache, especially if the teeth are decayed, then the gums become swollen, a suppuration ensues, and the teeth fall out of their sockets one after another. The pain then ceases, the bone is laid bare, and the style shows the existence of necrosis on a more or less considerable surface of the bone. One half of the patients attacked with necrosis from phosphorus died of it, and when they do not, a deformity remains, which, moreover, renders mastication and articulation of sounds extremely difficult. A visit paid to a manufactory of lucifer matches will convince the most incredulous from the acrid stench which prevails every where of the dangerous effects of such a trade.

